# ATP synthesis at physiological nucleotide concentrations

**DOI:** 10.1038/s41598-019-38564-0

**Published:** 2019-02-28

**Authors:** Axel Meyrat, Christoph von Ballmoos

**Affiliations:** Department of Chemistry and Biochemistry, University of Bern, Freiestrasse 3, 3012 Bern, Switzerland

## Abstract

Synthesis of ATP by the F_1_F_0_ ATP synthase in mitochondria and most bacteria is energized by the proton motive force (*pmf*) established and maintained by respiratory chain enzymes. Conversely, in the presence of ATP and in the absence of a *pmf*, the enzyme works as an ATP-driven proton pump. Here, we investigate how high concentrations of ATP affect the enzymatic activity of the F_1_F_0_ ATP synthase under high *pmf* conditions, which is the typical situation in mitochondria or growing bacteria. Using the ATP analogue adenosine 5′-O-(1-thiotriphosphate) (ATPαS), we have developed a modified luminescence-based assay to measure ATP synthesis in the presence of millimolar ATP concentrations, replacing an assay using radioactive nucleotides. In inverted membrane vesicles of *E. coli*, we found that under saturating *pmf* conditions, ATP synthesis was reduced to ~10% at 5 mM ATPαS. This reduction was reversed by ADP, but not P_i_, indicating that the ATP/ADP ratio controls the ATP synthesis rate. Our data suggests that the ATP/ADP ratio ~30 in growing *E. coli* limits the ATP synthesis rate to ~20% of the maximal rate possible at the applied *pmf* and that the rate reduction occurs via product inhibition rather than an increased ATP hydrolysis rate.

## Introduction

Life is energetically expensive and mostly paid in adenosine triphosphate (ATP). In all aerobic organisms, reducing equivalents from the oxidative breakdown of nutrients are converted to ATP in a process termed oxidative phosphorylation. Located in the cytoplasmic membrane of bacteria or the inner membrane of mitochondria, the members of the respiratory chain create and maintain a proton motive force (*pmf*) by proton coupled electron transfer reactions, e.g. from NADH to oxygen. The *pmf*, composed of a proton gradient (ΔpH) and a membrane potential (Δψ), energizes the rotary mechanism of the F_1_F_0_ ATP synthase that catalyzes the synthesis of ATP from its substrates ADP and P_i_ (for a review, see^1–4^). The F_1_F_0_ ATP synthase, which in most organisms can also work as an ATP driven proton pump, is composed of the membrane embedded F_0_ part and the soluble F_1_ part and can be divided into a stator and a rotor part. During ATP synthesis, when the *pmf* is high, protons are transported through the interface of subunit a and the c-ring converting the *pmf* into mechanical rotational energy. The c-ring is connected with subunits ε and γ forming the rotor, which transmits the rotary energy to the catalytic sites in the α_3_β_3_ headgroup. In the absence of a *pmf*, the enzyme can hydrolyze cellular ATP to establish an electrochemical gradient (the enzyme rotates in the opposite direction). This hydrolytic function of the F_1_F_0_ ATP synthase is vital for some bacteria under anaerobic conditions to maintain the membrane potential, when substrate level phosphorylation is the only source of ATP.

In order to minimize wasteful ATP hydrolysis, organisms have evolved different regulatory mechanisms, such as the inhibitory proteins IF1 in mitochondria^5,6^. A similar function has been attributed to the ζ subunit of the F_1_F_0_ ATP synthase of *Paracoccus denitrificans*, but is currently debated^7^. In other bacteria such as B*acillus* PS3 and *E. coli*, the ε subunit has been proposed to ratchet the rotor in the synthesis direction^8^ by extending its C-terminal domain along the γ subunit^9–12^, a conformational change that might be controlled by ATP binding in some bacteria^13^. An alternative mechanism was suggested recently, in which the inhibitory part of ε works in a similar fashion as IF1 in mitochondria: by blocking both ATP synthesis and hydrolysis in absence of a *pmf* and being released upon establishment of the *pmf* ^12,14^. A further role has been attributed to the ε subunit in the coupling of F_1_ reactions and F_0_ proton pumping^8,9,15–17^. In addition to these mechanisms, all F_1_F_0_ ATP synthases show a decreased ATP hydrolysis rate in the presence of high ADP concentrations, a phenomenon known as the MgADP-inhibition of ATP hydrolysis^18,19^. During MgADP inhibition, the enzyme is in a locked state, from which it can be reverted by e.g. the *pmf*. In some bacteria like *E. coli*, MgADP inhibition is less pronounced and the threshold *pmf* for re-activation lowered^20,21^. Taken together, these mechanisms prohibit complete cellular depletion of ATP in the absence of a sufficient *pmf*.

In contrast to ATP hydrolysis that can be investigated in detergent solution or even with the F_1_ part only, ATP synthesis experiments require the presence of a proton tight membrane and a *pmf*. Quantitative ATP synthesis experiments have been performed with a variety of biological material such as intact mitochondria, submitochondrial particles, chloroplasts, bacteria and inverted bacterial membranes, and purified F_1_F_0_ ATP synthase reconstituted into liposomes^22–27^. Depending on the used system, the *pmf* was established by natural proton pumps^23,28^ or an acid-base treatment in combination with a potassium/valinomycin diffusion potential to mimic the membrane potential^25,29,30^. Different methods to quantify ATP have been applied, including the following four. *(I)* The most popular and sensitive technique is based on the luciferase/luciferin couple, which in the presence of oxygen and ATP emits light that can be detected by a luminometer (for a review, see^31^). The technique is very sensitive and is applied to determine the total ATP content from all types of samples. However, it cannot be used to measure the synthesis of newly formed ATP in the presence of physiological concentrations of background ATP (mM) which is ~1000-fold too high. Consequently, very little to no ATP is present in kinetic ATP synthesis measurements that are followed by this method. *(II)* The problem is circumvented by the second method, where radioactively labeled ^32^PO_4_^2−^ or ^33^PO_4_^2−^ is added to the solution. Newly synthesized ATP will incorporate radiolabeled phosphate, while the already present ATP is not radioactive. After separation of excess labeled phosphate by extraction, the newly formed ATP can be quantified via autoradiography or scintillation counting. The drawbacks are the use of radioactive substances and the laborious workup that prohibits continuous measurements. *(III)* The third method is based on the conversion of produced ATP by coupled enzymatic reactions that can be detected by spectroscopy, e.g. addition of D-glucose in the presence of hexokinase and subsequent dephosphorylation by glucose-6-phosphate dehydrogenase in the presence of NAD(P) that is reduced to NAD(P)H and can be followed spectroscopically. Similar to luciferase, this method cannot discriminate between present or freshly synthesized ATP and is less sensitive than the other methods. (IV) A further and more indirect technique originally developed by Chance and colleagues^22^ to quantify ATP synthesis is to measure the alkalization in the surrounding buffer solution during ATP synthesis. During ATP formation from ADP and P_i_, a water molecule is formed that requires uptake of a proton from solution that can be measured either by a pH electrode or a pH sensitive dye such as phenol red. This approach is insensitive towards high concentrations of nucleotides and has successfully been used. However, it comes with limitations that have to be carefully addressed, such as low buffering capacity, limited pH range, and contribution of other proton dependent processes^32^.

In the present work, we describe a modification of the luciferase/luciferin technique that allows rapid continous measurement of the ATP synthesis rate with a high time resolution at physiological (millimolar range) concentrations of ATP. The principle is based on the properties of a modified ATP, adenosine-5′-O-(1-thiotriphosphate), or ATPαS, which is a hydrolytic substrate for the F_1_F_0_ ATP synthase, but is not detected by the luciferin/luciferase system. With the developed method, we were able to measure and compare the ATP synthesis in inverted *E. coli* membrane vesicles as a function of ADP, ATPαS, and inorganic phosphate (P_i_) concentrations. We observe that physiological concentrations of ATP (5 mM), under the form of ATPαS, reduce the ATP synthesis rate to ~10%, and that this rate can be restored using elevated ADP concentrations, but not P_i_, suggesting that the ATP synthesis is controlled by the ATP/ADP ratio and not by the ATP/(ADP∙P_i_) ratio. The applicability of the technique to more isolated systems was confirmed in ATP synthesis experiments in liposomes containing purified *bo*_3_ oxidase and ATP synthase from *E. coli*.

## Results

### A modified luciferin/luciferase assay

The widely used reaction of D-luciferin with ATP, catalyzed by the luciferase from firefly, suffers from the drawback that it cannot discriminate between already present and freshly synthesized ATP. In addition, the high sensitivity of the assay impairs the use of ATP concentrations higher than a few micromolar, which is ~1000-fold less than what is found *in vivo*. The luciferase first catalyzes the binding of D-luciferin to the α-phosphate of ATP-Mg^2+^, forming PP_i_-Mg^2+^ and luciferyl-AMP, which is subsequently oxidized by oxygen to form AMP, CO_2_ and oxyluciferin in an excited state that emits a photon while returning to the ground state (Supplementary Fig. S1, for a review, see^33,34^). ATPases like the F_1_F_0_ ATP synthase, however, cleave ATP between the β- and the γ-phosphate, leaving ADP and P_i_ behind. We therefore reasoned that the use of an α-phosphate modified ATP prohibits the reaction with the luciferase but allows the reaction with ATPases. Indeed, commercially available ATPαS, in which the double-bonded oxygen on the α-phosphate is substituted for a sulfur, has been shown not to be a substrate for the luciferase in the deoxy form^35^, but is hydrolyzed by ATPases including the chloroplast F_1_F_0_ ATP synthase^36^ and the F_1_ ATPase from *Bacillus* PS3^37^.

**Figure 1:**
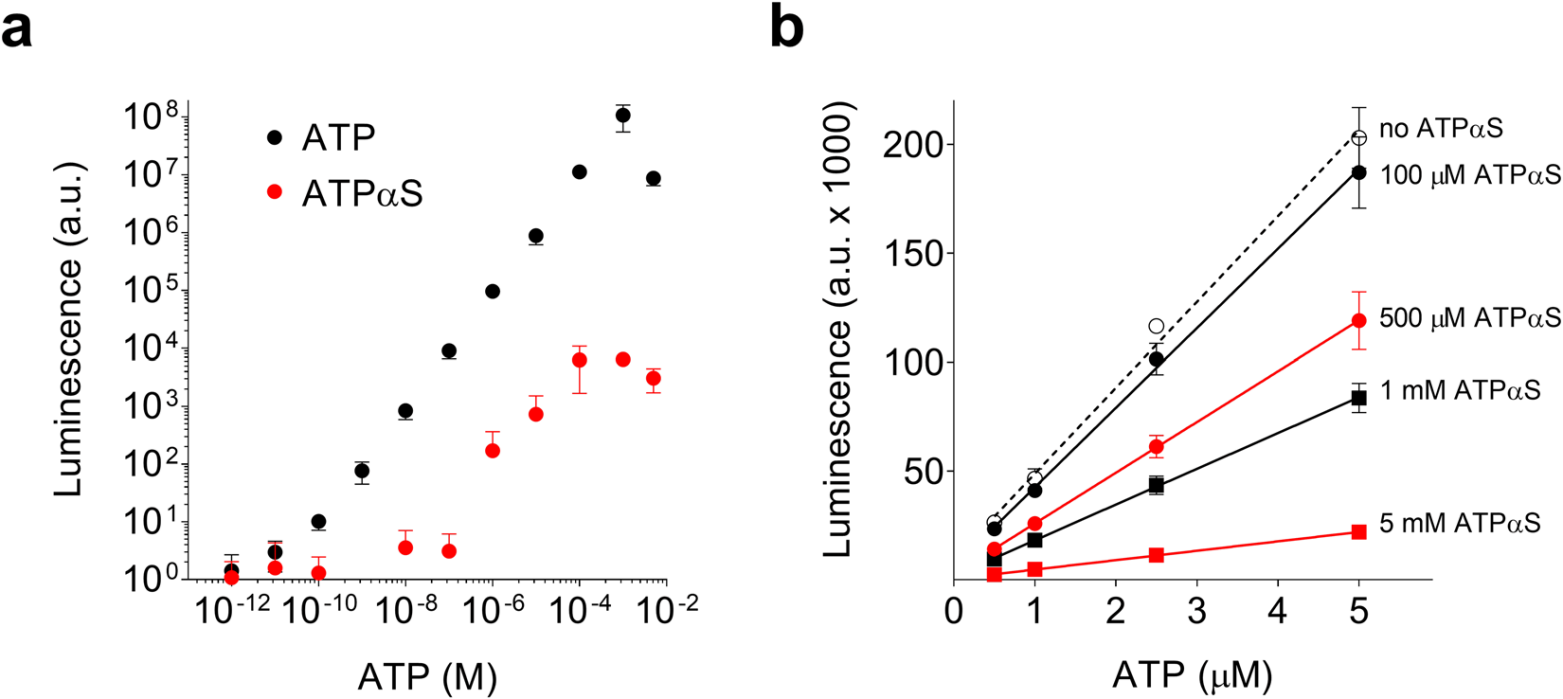
The reaction of ATPαS vs. ATP with luciferin/luciferase. (**a)** Varying amounts of ATP (•) and (ATPαS, ) were mixed with 2 µl luciferin/luciferase (prepared according to the manufacturer), filled up to 100 µl with buffer and measured in triplicate in a tube-type luminometer. (**b**) Dependency of the luminescence on ATP in the presence of increasing concentrations of ATPαS. The high concentrations of ATPαS used in our experiments quench luminescence to some extent and thus standardization by addition of a small known concentration of ATP is needed (as shown in Fig. 3b). Under all indicated ATPαS concentrations, addition of 0.5, 1, 2.5 and 5 µM ATP resulted in a linearly dependent luminescent signal. The measurements were done in triplicate and linear regressions are shown.

We tested this hypothesis experimentally by comparing the reaction of ATP and ATPαS with the firefly luciferase/luciferin supplied as a mixture in a commercial kit (CLS II, Roche) that is used by many to detect ATP synthesis. A typical amount of the assay was mixed with buffer and varying amounts of normal ATP or ATPαS were added to reach a final nucleotide concentration ranging from 10^−12^ M up to 5 × 10^−3^ M. The luminescence produced by the reaction of the luciferase reagent with ATP spanned from ~10° to ~10^8^ arbitrary units (a.u.) between 1 pM and 5 mM whereas ATPαS yielded <10^4^ a.u. at 5 mM (Fig. 1a), representing ~0.01% of the ATP signal response. The observed signal in the reaction with ATPαS is due either to reaction with ATPαS (with a high K_m_ or low V_max_) or to contaminating ATP in the ATPαS preparation. We also found that ATPαS concentrations above 100 µM decreased the overall luciferase signal (<50% at 1 mM), probably due to competition of ATPαS with ATP for the luciferase binding site. Experimentally, this effect is addressed by adding a known concentration of ATP (2.5 µM in our experiments) to relate the arbitrary units of the luminescent signal to the concentration of ATP for the quantification of ATP production. Figure 1b shows that the luminescence signal was linear in the relevant ATP concentration range at all tested ATPαS concentrations.

### ATPαS is a substrate for the *E. coli* F_1_F_0_ ATP synthase

**Figure 2:**
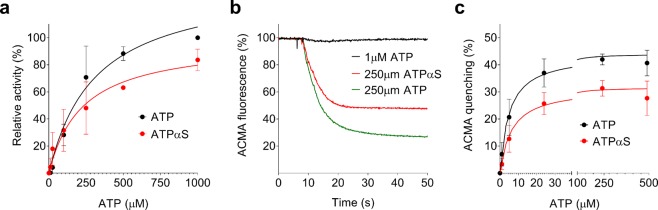
The reaction of ATPαS with the *E. coli* F_1_F_0_ ATP synthase. (**a**) Varying amounts of ATP (•) and (ATPαS, ) were incubated with purified *E. coli* F_1_F_0_ ATP synthase and ATP hydrolysis was measured using a phenol red assay. Shown are average values and standard deviation from three measurements. (**b**) ATP and ATPαS hydrolysis-driven proton pumping by *E. coli* F_1_F_0_ ATP synthase in inverted membrane vesicles was followed using ACMA fluorescence quenching. Shown are typical traces, here for 1 µM ATP (black), 250 µM ATPαS (red) and 250 µM ATP (green). (**c**) Experiment like b, but varying amounts of ATP (•) and ATPαS() were used, taking into account the initial ACMA quenching (within 5 sec) of the different measurements. Shown are average values and standard deviation from three measurements.

ATP and ATPαS were compared as substrates for hydrolysis by the *E. coli* F_1_F_0_ ATP synthase in detergent solution (Fig. 2a). Using different concentrations of either nucleotide, the ATP hydrolysis rates were determined using phenol red to detect proton consumption during ATP hydrolysis^38^. The measured ATPαS hydrolysis rate (apparent K_m_ 226 µM, ~21 ATP s^−1^ enzyme^−1^ at 1 mM) was 84% of that of ATP at the maximum tested substrate concentration (apparent K_m_ 280 µM, ~25 ATP s^−1^ enzyme^−1^ at 1 mM). We also compared the ability of ATP and ATPαS to elicit transmembrane proton pumping, i.e. establishment of a *pmf*, with ACMA quenching in inverted membrane vesicles (IMV) of *E. coli* cells (Fig. 2b,c). Both substrates energized proton pumping effectively, indicating that ATPαS hydrolysis by the *E. coli* F_1_F_0_ ATP synthase does not interfere with the rotational mechanism of the enzyme. The lower apparent K_m_ values for proton pumping could be attributed to the different type of preparation (purified enzyme vs. inverted membrane vesicles). Proton pumping was accelerated ~2.5-fold in the absence of a membrane potential (1 µM valinomycin added, Supplementary Fig. S2), indicating that the IMVs were reasonably tight. A lower maximum (~75%) initial quenching rate and total quenching was observed with ATPαS compared to ATP. Both findings can be explained by the decreased ATP hydrolysis rate described above, as the ACMA quench signal is dependent on both the rate of proton pumping and the rate of spontaneous proton leakage through the membrane.

### ATP synthesis kinetics of inverted *E. coli* vesicles at physiological ATP concentrations

**Figure 3:**
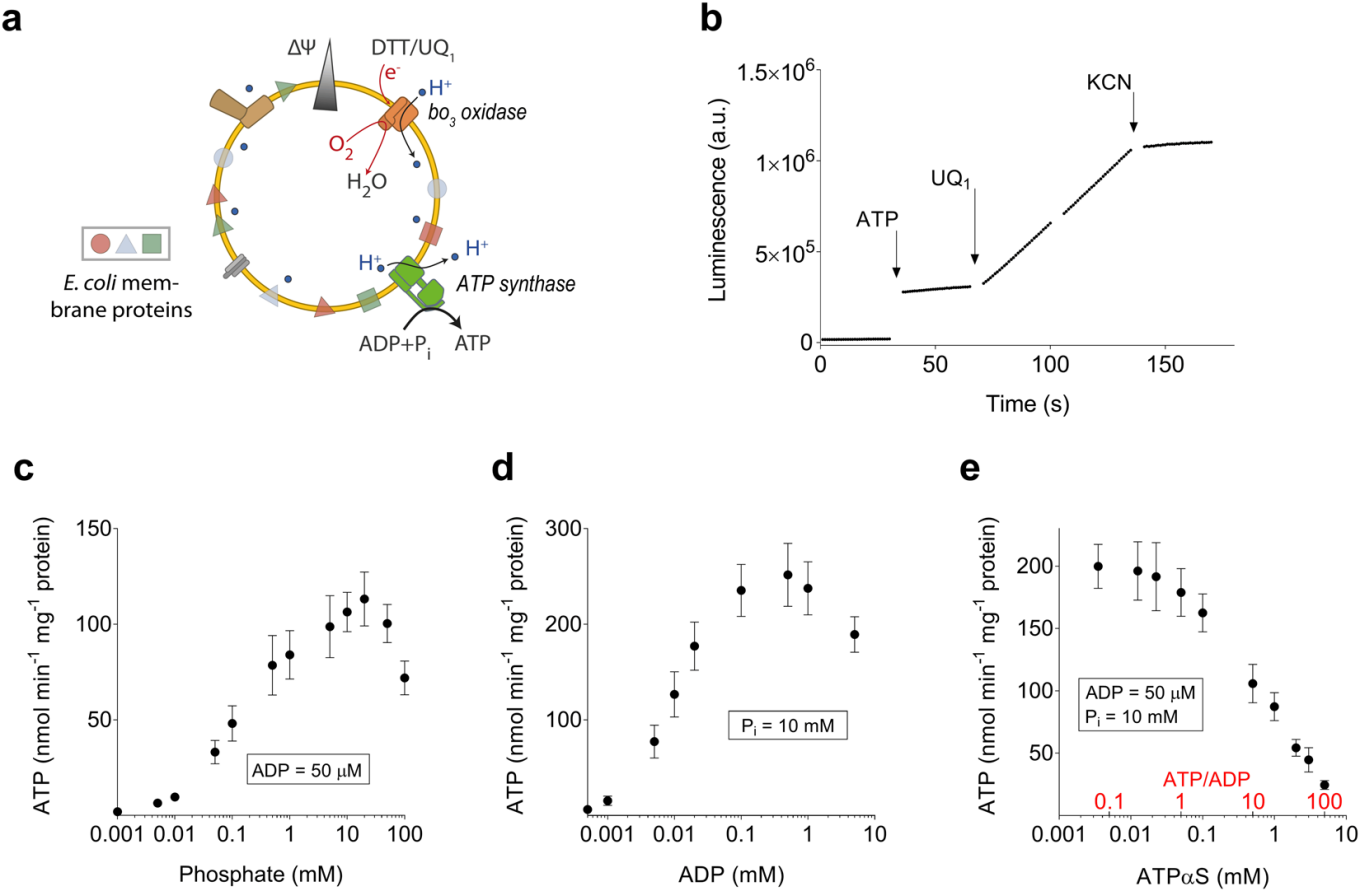
Respiratory chain driven ATP synthesis in inverted membranes of *E. coli*. **(a)** Cartoon illustrating the experimental setup. Inverted membrane vesicles of *E. coli* were energized using DTT and ubiquinone Q_1_ to initiate inward proton pumping of the quinol *bo*_3_ oxidase, generating a *pmf* (see Supplementary Fig. S4). ATP synthesis was catalyzed by the F_1_F_0_ ATP synthase with its F_1_ head directed toward to the outside and detected using a luminescent based assay. (**b)** Raw data from continuous measurement of ATP synthesis in IMVs using luminescence in the presence of 50 μM ADP and 10 mM P_i_. Proton pumping by the *bo*_3_ oxidase was supported by DTT as electron donor and ubiquinone Q_1_ (UQ_1_) as electron mediator. Addition of the cytochrome oxidase inhibitor potassium cyanide (KCN) stops ATP synthesis. The arrows indicate the different point of additions. From such data, the slope of the linear range and the ATP standard addition were used to calculate ATP synthesis rates in the following figures. Luminescence values were for periods of 30 s, allowing addition of reagents when needed (indicated by short gaps in the trace). (**c)** Dependency of ATP synthesis rate from the P_i_ concentration in the presence of 50 μM ADP and in the absence of ATP. (**d**) Dependency of ATP synthesis rate from the ADP concentration in the presence of 10 mM P_i_ and in the absence of ATP. (**e)** Dependency of the ATP synthesis rate from the ATPαS concentration in the presence of 50 μM ADP and 10 mM P_i_. The ATP/ADP ratio is indicated in red.

In this work, we applied the novel method described above to follow ATP synthesis in the presence of high ATP concentration and high *pmf*, reflecting the physiological conditions of growing bacteria. As a model system, we used inverted membrane vesicles (IMVs) of *E. coli*, produced by a French Press^39^. In contrast to mitochondria, *E. coli* membranes do not contain nucleotide and phosphate carriers that control the availability and distribution of their substrates in the mitochondrial matrix. In IMVs, the orientation of all proteins is homogenously inverted^40^, exposing the F_1_ part of the F_1_F_0_ ATP synthase to the buffer solution, allowing to readily manipulate the relevant substrates ATP, ADP and P_i_. For prospective studies investigating mutant variants, we used an *E. coli* F_1_F_0_ ATP synthase knockout strain (DK8), transformed with the plasmid pBWU13, constitutively expressing F_1_F_0_ ATP synthase. This combination has been shown to yield F_1_F_0_ ATP synthase overexpression when grown in minimal media using glycerol as carbon source^41^. If grown in rich media, however, F_1_F_0_ ATP synthase production levels were comparable to a strain BL21 with chromosomally encoded F_1_F_0_ ATP synthase, as judged by Western blot (Supplementary Fig. S3). As complex I is expressed predominantly under anaerobic conditions^42^, we have used dithiothreitol (DTT) and ubiquinone Q_1_ as synthetic electron donor and acceptor, respectively, to energize the quinol oxidase *bo*_*3*_ as the primary proton pump to establish a maximal *pmf* (Fig. 3a). The required amount of ubiquinone Q_1_ (≥20 µM) to obtain maximal ATP synthesis was titrated (Supplementary. Fig. S4) and in all measurements, a ubiquinone Q_1_ concentration of 80 µM was used to ensure sufficient *pmf* for maximal ATP synthesis. Figure 3b shows a typical continuous ATP synthesis measurement in IMV of *E. coli* as described^26,43^, with almost no free ATP present (a defined concentration of ATP was added prior to measurement to quantify the signal), that was started by the addition of 80 µM ubiquinone Q_1_. ATP synthesis was immediately observed as a linear increase of the luminescence signal and was followed for at least 30 s. Addition of 400 nM potassium cyanide, a potent inhibitor of the *bo*_3_ oxidase, rapidly blocks ATP generation. Similarly, <10% ATP synthesis was observed in the presence of the proton ionophore CCCP (10 µM). Using 2 mM DTT and 80 µM ubiquinone Q_1_ as electron donor, the ATP synthesis rate was about 1.5 to 2-fold higher compared to energization with 500 µM NADH, both in the DK8/pBWU13 and BL21 strains (Supplementary Table 1).

In a first set of experiments, we characterized our experimental system by varying concentrations of inorganic phosphate (Fig. 3c) and ADP (Fig. 3d). Apparent K_m_ values for ADP and phosphate were 11 µM and 104 µM, respectively. Under fully saturating conditions using 50 μM ADP and 10 mM phosphate, a maximal ATP synthesis rate of ~250 ± 50 nmol ATP min^−1^ mg^−1^ was obtained at 25 °C, which is in good accordance to a recent report for *E. coli* IMVs^7^. At maximal tested ADP (5 mM) and P_i_ (100 mM) concentrations, the ATP synthesis rate declined to ~70%.

Next, we tested the influence of increasing concentrations of ATPαS on the rate of ATP synthesis in the presence of a constantly high *pmf*. Keeping ADP (50 µM) and phosphate (10 mM) constant, we varied the ATPαS concentration between 1 µM and 5 mM, resulting in a distinct decrease in the synthesis rate. As depicted in Fig. 3e, ATP synthesis was maximal below <25 µM ATPαS (100%) but started declining beyond 50 μM ATPαS, reaching a residual rate of 10% to 20% in the physiologically relevant concentration range of 2 to 5 mM.

**Figure 4:**
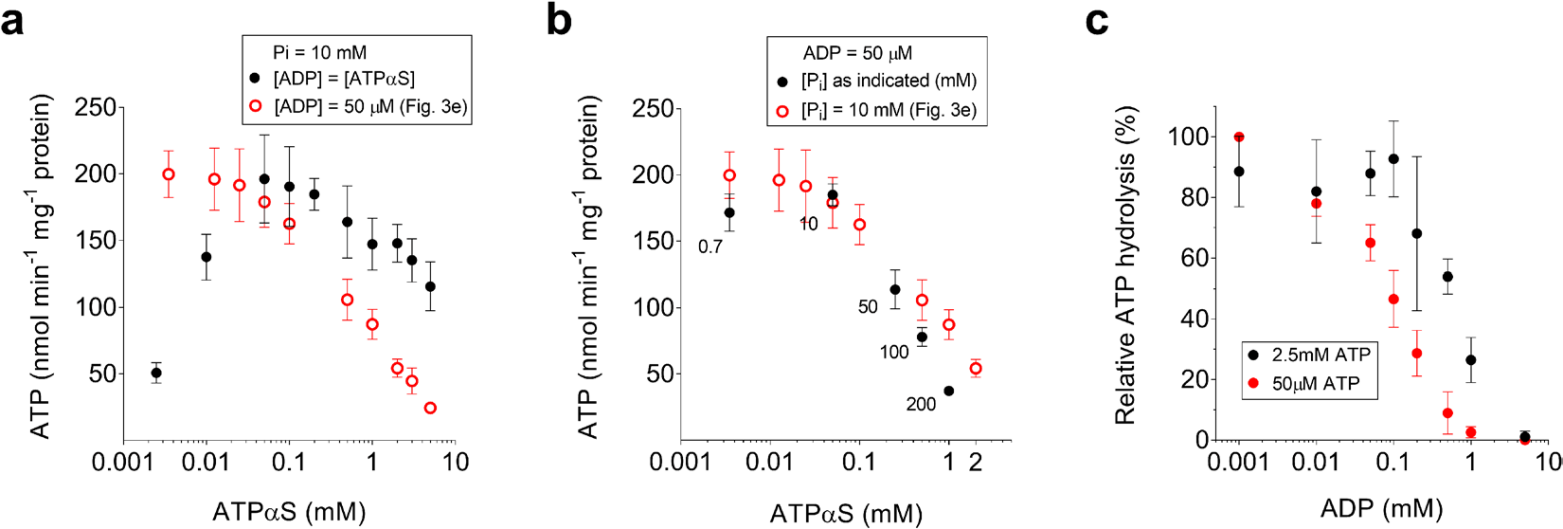
Dependency of ATP synthesis and hydrolysis on nucleotide concentration. The values of ATP synthesis rate at increasing adenosine 5′-O-(1-thiotriphosphate) (ATPαS) and ΔG (constant 50 μM ADP and 10 mM P_i_, Fig. 3e) are indicated as  for comparison. (**a)** An equimolar concentration of ADP was used to keep a constant ATP/ADP ratio of 1 and a ΔG of 40 kJ/mol, in the presence of 10 mM P_i_. (**b)** Like a, but a constant ATPαS/P_i_ ratio of 0.005 was used to keep constant a ΔG of 40 kJ/mol (P_i_ concentrations are indicated in millimolar below each circle), in the presence of 50 μM ADP. (**c)** ATP hydrolysis as a function of the ADP concentration, with an ATP concentration of 50 μM  or 2.5 mM (•). The activity was followed by measuring the initial ACMA fluorescence quenching (5 sec) during proton pumping.

To check whether the decrease in rate was due to the absolute ATPαS concentration or not, we investigated how the initial apparent mass-action ratio for the ATP synthesis reaction Γ’ = [ATP]/([ADP]∙[P_i_]) influences the ATP synthesis rate. At chemical equilibrium, where no net ATP synthesis takes place the mass-action ratio is equal to the equilibrium constant K^44^. In equilibrium thermodynamics, the Gibbs free energy change is defined by the equation

$${\rm{\Delta }}{\rm{G}}=-\,2.3RT\cdot {\mathrm{log}}_{10}({\rm{K}}/{\rm{\Gamma }}),$$

where R is the gas constant and T the temperature. While thermodynamic considerations cannot be used to predict kinetic rates, the data of Fig. 3e show that varying the initial Γ’ (100 M^−1^ at 50 µM ATPαS and 10^4^ M^−1^ at 5 mM ATPαS) directly affect ATP synthesis rates. It was thus interesting to observe whether the effect caused by increased ATPαS concentrations could be reverted by increasing ADP or phosphate simultaneously, thus leaving Γ’ constant (Γ’ = 100 M^−1^). First, we tested this by increasing both ADP and ATPαS concentrations in parallel at a fixed P_i_ concentration (10 mM) to keep Γ’ = 100 M^−1^ and ATPαS/ADP = 1 constant (Fig. 4a). If ATPαS and ADP were below 50 µM, non-maximal activity was observed due to non-saturating ADP levels (compare to Fig. 3d). Maximal ATP synthesis rate was observed between 50 and 200 μM nucleotides (100%, 196 ± 33 nmol ATP min^−1^ mg^−1^), and slowly decreased to 60% (116 ± 18 nmol ATP min^−1^ mg^−1^) at 5 mM ATPαS and ADP. Considering the decrease of activity by 5 mM ADP itself (75%, see Fig. 3d), ATP synthesis was recovered to ~85%, demonstrating that increasing ADP concentrations can revert the effect observed above. In a second scenario, we adjusted ATP and P_i_ in parallel in the presence of a fixed ADP concentration (50 µM), to keep Γ’ = 100 M^−1^ and ATP/P_i_ = 0.005 constant. The data from Fig. 4b show that in contrast to ADP, inorganic phosphate cannot reverse the inhibitory effect of ATPαS. The curve decreases more than the one seen in Fig. 3e, declining from a maximum of 185 ± 8 nmol ATP min^−1^ mg^−1^ at 50 μM ATPαS (ATP/ADP = 1) to 37 ± 3 nmol ATP min^−1^ mg^−1^ (20 ± 3% of maximum observed rate) at 1 mM ATPαS (ATP/ADP = 20). This pronounced decrease is probably due to the inhibitory effect observed at high phosphate concentration (Fig. 3c).

We have repeated the crucial experiments also with proteoliposomes containing F_1_F_0_ ATP synthase and a proton pump prepared as described^28^. To this end, the purified *E. coli* F_1_F_0_ ATP synthase and *bo*_3_ oxidase were co-reconstituted into liposomes made of 4:1 DOPC:DOPG (w/w). Proton pumping was initiated with DTT/Q_1_ and ATP synthesis was followed as described for the inverted membrane vesicle. First, ATP synthesis rates in the presence of 50 µM ADP, and either 10 µM or 2 mM ATPαS were measured. As depicted in Supplementary Fig. S5, ATP synthase rate dropped to ~6% in the presence of 2 mM ATPαS, making the inhibitory effect even stronger than with IMVs (~18%) at the same ATPαS concentration. In the presence of 2 mM ADP, the activity was recovered to ~50% (75% in the IMVs). Taken together, the measurements support our findings with the membrane vesicles and reinforce the notion that the effect is directly connected to the F_1_F_0_ ATP synthase.

## Discussion

### ATPαS can be used as a substrate analogue for ATP hydrolysis in the *E. coli* F_1_F_0_ ATP synthase

In order to allow continuous detection of ATP synthesis by a luciferase/luciferin assay in the presence of high ATP concentrations, we have used a modified ATP analogue that is not detected by the luciferase system but is a suitable substrate for ATP hydrolysis with similar kinetic properties as ATP. We show that the sulfur-substituted alpha phosphate indeed barely reacts with luciferin. The observed signal increase upon rising ATPαS concentration (~10^4^ times lower than ATP) is due either to a very slow reaction of ATPαS with luciferin or to slight ATP contamination in the ATPαS preparation. ATPαS binding to luciferase/luciferin as a competitive inhibitor is likely, as the overall signal for a constant amount of ATP is decreasing with increasing concentrations of ATPαS. Technically, this effect was compensated by the addition of a precisely known, suitable amount of ATP for every measurement to quantify the luminescence signal. In contrast to its reaction with luciferin/luciferase, ATPαS behaved similarly to ATP when used to drive ATP hydrolysis with the *E. coli* F_1_F_0_ ATP synthase. As with the F_1_F_0_ enzyme in chloroplasts and the isolated F_1_ part of the ATP synthase of *Bacillus* PS3, ATP hydrolysis and proton pumping kinetics with the *E. coli* enzyme were comparable. In the reaction with ATPαS, V_max_ was ~75% of the one found for ATP and both had a similar K_m_’ of ~200–300 µM in the absence of ADP. Earlier, Löbau *et al*. reported an apparent K_m_(MgATP) = 140 µM for *E. coli* F_1_F_0_ ATP synthase^45^. Data for ATPαS with intact F_1_F_0_ ATP synthase is missing, but in elegant single-molecule experiments following the rotation of the gamma subunit, Noji and coworkers have found an apparent K_m_ (91 µM) with the purified F_1_ ATPase from *Bacillus* PS3 lacking the regulatory epsilon subunit^37^. In comparison to published values, the Km values reported here were ~2-fold higher, which is likely due to different assay conditions (e.g. buffer, Mg^2+^ concentration) and different enzyme preparations from different organisms. However, given the similar behavior within our preparation, it was concluded that ATPαS can be used to replace background ATP in ATP synthesis experiments. In the past, ATP synthesis experiments in the presence of high ATP were performed following the incorporation of radioactive ^32^PO_4_^2-^ or ^33^PO_4_^2-^ into newly formed ATP.

### The ATP synthesis rate is strongly reduced by an increasing ATP/ADP ratio

The physiological nucleotide concentration ratio (ATP/ADP ~20–1000) strongly favors ATP hydrolysis (ATP/ADP at equilibrium is ~10^−5^). However, the presence of respiratory proton pumps (e.g. *bo*_3_ oxidase in *E. coli*) generating and maintaining a *pmf* are able to shift the net reaction towards ATP synthesis. Typical *in vitro* ATP synthesis studies nevertheless employ conditions where no ATP is present due to the incompatibility with the luciferase assay, while ADP is used in excess to maximize the observed rates. In this report, we describe the use of ATPαS to overcome this limitation and have investigated the effect of cellular concentrations of ATPαS (as a substrate analogue of ATP) on the enzymatic reaction of the *E. coli* F_1_F_0_ ATP synthase directly. Similar measurements have been performed in intact mitochondria, but the interpretation of results is complicated by the presence of *pmf* driven phosphate and nucleotide carriers from the SLC25 family that transport these substrates from the bulk to the site of ATP synthesis in the mitochondrial matrix. Consequently, the different substrates added to the outside affect the F_1_F_0_ ATP synthase only indirectly. By contrast, no such transporters are present in *E. coli* and the complete inversion of protein orientation in IMVs allows direct access of the experimentally added substrates ATP, ADP and P_i_ to the catalytic sites in the F_1_ part of the ATP synthase.

The influence of the cytoplasmic ATP/ADP ratio on the ATP synthesis rate had been investigated with intact mitochondria by e.g. Küster *et al*.^24^ and LaNoue *et al*.^46^. Küster *et al*. report that the extra-mitochondrial ATP/ADP ratio controls the ATP synthesis rate, dropping steeply after a ratio of ~5 with essentially no ATP synthesis at a ratio of 100. LaNoue *et al*. report that ATP synthesis and ATP hydrolysis are only in equilibrium at low *pmf*, while at high *pmf* values, the increased net ATP synthesis is due to nearly blocked ATP hydrolysis rather than an increased ATP synthesis rate. Both studies confirm a kinetic control of the ATP synthesis rate with the ATP/ADP translocase being the controlling module^46^. Omitting the added complexity of the ATP/ADP translocase, Ferguson and colleagues investigated cells and membranes of the gram negative bacterium *Paracoccus denitrificans*^47^. In their studies, they find that membrane vesicles of *P. denitrificans* are not capable of ATP hydrolysis, neither under high or low *pmf* conditions, and conclude that the kinetic control of ATP synthesis is an intrinsic property of the F_1_F_0_ ATP synthase. There is an ongoing discussion to determine if the backwards reaction in *P. denitrificans* is blocked by the ζ subunit or not^6,7,48^, or if the inhibitory effect is caused by Mg-ADP inhibition, as proposed earlier^49^.

With the present study, we can add information to the situation in *E. coli*, which represents a mixed situation. Like *P. denitrificans*, no ATP/ADP translocase is present, allowing a more direct investigation of the properties of the F_1_F_0_ ATP synthase. However, in contrast to *P. denitrificans*, membranes of *E. coli* show no respiratory coupling and the F_1_F_0_ ATP synthase is fully active in ATP hydrolysis direction^32,50,51^. In our measurements with a constant amount of ADP and phosphate, an increasing ATPαS concentration (and thus ATP/ADP ratio) provokes a sharp decrease of ATP synthesis over an ATP/ADP ratio of 1, with 50% activity at ratio of ~20 (1 mM ATPαS) and <10% activity at a ratio of 100 (5 mM ATPαS), which is in good agreement with the data from mitochondria. As the *pmf* in our experiments is unchanged (no notable effect of increasing nucleotide concentrations on respiratory activity were found), the data indicates that the observed effect is either due to product inhibition by the presence of ATPαS or by an increased rate of ATP hydrolysis. Since our assay directly detects newly formed ATP with a very high time resolution and an immediate decrease in ATP synthesis rate is observed, we can confirm that a direct competition of ATPαS and ADP indeed takes place. We cannot rule out an increased ATP hydrolysis rate, but the effective ATPαS concentrations (>1 mM) are well above the apparent K_m_ of ATP for ATP hydrolysis, which however could be increased in the presence of a *pmf*. In similar experiments, where ADP was raised in parallel to keep constant the ATPαS/ADP ratio and mass-action ratio (Γ’ = 100 M^−1^), the inhibitory effect of ATPαS was only mildly observed, suggesting that the competition of ADP and ATPαS indeed control the rate of ATP synthesis. A more detailed interpretation of these results is complicated by the presence of three catalytic sites that have different affinities for ATP and ADP depending on their conformation state (tight, loose, and empty)^52^. Finally, by varying the ATP and P_i_ concentrations in parallel, resulting in the same Γ’ = 100 M^−1^, but increasing the ATP/ADP ratio, we found that a variation of the phosphate concentration could not prevent the inhibitory effect, indicating that ATP synthesis is not controlled by the phosphorylation potential variable [ATP]/([ADP]∙[P_i_]), but rather by the ATP/ADP ratio. The inhibitory effect of ATPαS at higher phosphate concentrations was even more pronounced (<5% residual activity at 5 mM ATPαS), which can be attributed to a slight inhibitory effect of P_i_ concentrations >100 mM.

In respiring *E. coli*, the ATP and ADP concentrations have been found to be ~3.5 mM and 0.12 mM, respectively, yielding an ATP/ADP ratio of ~30^53^. From our measurements, we can thus assume that despite saturating *pmf*, the *E. coli* synthesizes ATP with a rate of only ~20% of v_max_. Etzold *et al*. determined a maximal turnover rate of 270 ATP s^−1^ per ATP synthase in *E. coli* at 37 °C^54^. However, in their measurements, 95% of the ATP synthases were blocked to maximize the available driving force per F_1_F_0_ ATP synthase. Furthermore, their experiments were performed in the presence of 1 mM ADP and the total absence of ATP (ATP/ADP = 0). It can thus be estimated that the ATP synthesis rates is only 5 to 10 ATP s^−1^ per F_1_F_0_ ATP synthase in growing *E. coli*. The observed necessary concentrations of ADP >1 mM to restore maximal ATP synthesis are not physiologically relevant. However, the nearly universal ATP hydrolysis inhibition mechanism by Mg-ADP^52,55–59^ could be responsible for the apparent absence of ATP hydrolysis in our experiments. We have thus measured the inhibitory effect of ADP on proton pumping on the same vesicles as used for ATP synthesis. The data from Fig. 4c show that the apparent K_i_ of ADP increases from ~100 µM to ~400 µM in the presence of 50 µM ATP or 2.5 mM ATP, respectively. An even higher K_i_ is expected during ATP synthesis under high *pmf*, that has been shown to counteract ADP inhibition of ATP hydrolysis^19,57^, and hence being considerably higher compared to the ADP concentrations found in growing cells (~120 µM)^53^. ADP concentrations >400 µM, however will be easily reached when the *pmf* is low and ATP is converted to ADP, and the mechanism thus serves as effective protection against cellular depletion of ATP. Finally, our results seem to be independent of the reported inhibition of ATP hydrolysis by the ε subunit, as for the *E. coli* enzyme it has been shown to be absent in the presence of a *pmf* and independent of the ATP concentration^14^. In the future, it will be interesting to investigate, if ATP synthesis in other organisms than *E. coli* is similarly dependent on the ATP concentration.

## Concluding Remarks

In this work, we have introduced a novel, alternative, non-radioactive method to measure ATP synthesis *in vitro* under physiological nucleotide concentrations and high *pmf*, using ATPαS as a replacement for ATP. The method allows to selectively measure newly produced ATP and is unaffected by the presence of high concentrations of ATPαS. The luciferase setup allows rapid and continuous measurements, avoiding the necessity of taking samples at distinct time points, thus considerably increasing time resolution in measurements.

We have applied this method to inverted membrane vesicles of *E. coli* and proteoliposomes containing F_1_F_0_ ATP synthase and *bo*_3_ oxidase and find that the ATP/ADP ratio, but not ATP/(ADP∙P_i_), controls the rate of ATP synthesis. The effect is based on a direct competition between of ATPαS and ADP for the catalytic binding sites. From this, we can conclude that the physiological ATP concentration prevents a maximal ATP synthesis rate despite saturating *pmf* conditions. Finally, our results suggest that Mg-ADP inhibition of ATP hydrolysis is not relevant to maximize ATP synthesis rates in *E. coli*, but is an effective tool to prevent total loss of cellular ATP during energy starvation.

## Material and Methods

Chemicals and kits were purchased from Sigma-Aldrich and Thermo Scientific if not otherwise stated.

### Growth conditions of bacterial cells

*E. coli* strain DK8, lacking the *unc* operon coding for its ATP synthase, was transformed with a pBWU13 plasmid and grown at 37 °C in LB-Lennox medium containing 50 μg/ml ampicillin.

### Preparation of Escherichia coli DK8:pBWU13 inverted membrane vesicles

The following procedure was performed at 4 °C. Bacterial cells were suspended in 10 ml/g HMG buffer (20 mM Hepes pH 7.5, 2 mM MgCl_2_, and 10% glycerol), supplemented with a tiny spatula tip of Pefabloc serine protease inhibitor, phenylmethanesulfonyl fluoride and DNAse I, and broken with a French pressure cell press or a Microfluidizer. Unbroken cells were collected by centrifugation at 8000 *g* for 10 min. The supernatant containing the membrane vesicles was centrifuged at high speed (200′000 *g*) for 1 h, resuspended in 5 ml HMG supplemented with 0.5% cholate per gram of initial cells, to remove proteins loosely associated with the membrane, and centrifuged again at high speed. The membrane vesicles were resuspended in HMG and centrifuged a third time at high speed to remove traces of cholate. They were then resuspended in HMG to a concentration of 10 mg/ml total proteins (quantified with a bicinchoninic acid assay), aliquoted and flash-frozen with liquid nitrogen for storage at −80 °C.

### Purification of F1F0 ATP synthase from *E. coli*

The F_1_F_0_ ATP synthase of *E. coli* strain DK8:pBWU13 was purified as previously described^26^.

### ATP hydrolysis assay by ACMA quenching

With this method we indirectly measure the hydrolytic activity of the F_1_F_0_-ATPase by following the establishment of the resulting proton gradient, using the self-quenching property of the fluorescent dye 9-Amino-6-Chloro-2-Methoxyacridine (ACMA). The fluorescence of this membrane permeable dye quenches when a proton gradient forms across a lipid compartment^60^.

The fluorescence of the dye 9-Amino-6-Chloro-2-Methoxyacridine (ACMA) (excitation wavelength: 410 nm; emission wavelength: 480 nm) was monitored with a Cary Eclipse Fluorescence Spectrophotometer from Agilent Technologies. A cuvette with stirring magnet was filled with HMK buffer (10 mM Hepes-KOH pH 7.5, 1 mM MgCl_2_, 10 mM KNO_3_), 150 μg/ml inverted bacterial membrane vesicles and 2 μM ACMA in a final volume of 1 ml. The fluorescence baseline, taken as 100% fluorescence to determine the percentage of quenching, was measured and hydrolysis was started by the addition of 1–500 μM ATP or ATPαS. The pH gradient established by the ATP synthase quenched the ACMA dye proportionally to the hydrolytic activity and was eventually dissipated by the addition of 20 mM NH_4_Cl.

To assess the effect of valinomycin and the coupling of IMVs, a buffer containing 10 mM MOPS-KOH pH 7.5, 100 mM KCl, and 2.5 mM MgCl_2_ was used instead of HMK. The measurement was carried as above, with or without 1 μM valinomycin, and started with 500 μM ATP.

### ATP hydrolysis assay by phenol red spectrophotometry

Phenol red is a pH-sensitive dye which can be used to detect the dissociation of protons in the ATP hydrolysis reaction^38^, at pH > 7.2: ATP^4−^ + H_2_O ⇌ ADP^3−^ + P_i_^2−^ + H^+^ (see for example^61^).

Phenol red buffer (0.5 mM Hepes-KOH pH 8, 100 mM KCl, 4 mM MgCl_2_, 0.1 mM EDTA, 10% glycerol, 0.02% triton X-100) was mixed with 25 μM phenol red and desired concentrations of ADP, ATP and ATPαS, to a final volume of 1 ml. The pH was adjusted to 8 with KOH and the baseline absorbance was recorded at 557 nm with a Cary 60 UV-Vis spectrophotometer. 20 nmol HCl were used for standardization and 5 μg *E. coli* purified F_1_F_0_ ATP synthase were added to start the reaction.

### Luciferin/luciferase reaction with ATP versus ATPαS

The firefly enzyme luciferase drives an ATP-dependent reaction in two steps to produce light that is proportional to the amount of ATP present and can be measured with a luminometer.

To compare the reactivity of ATP *versus* ATPαS with the luciferin/luciferase system, 200 μg/ml luciferase reagent (containing luciferin, luciferase and magnesium) of an ATP Bioluminescence Assay Kit CLS II from Roche Applied Science was added to 50 mM Hepes-NaOH pH 7.5, 10 mM total phosphate and 3 mM MgCl_2_, and the baseline was measured. 1 μM to 5 mM ATP or ATPαS was then added and the luminescence measured.

### Luciferin/luciferase ATP synthesis assay

The assay was performed in clear 1.5 ml Eppendorf tubes in a final volume of 100 μl. 50 mM Hepes-NaOH pH 7.5 were used as buffer and the assay mixture contained 10 mM MgCl_2_, 200 μg/ml luciferase reagent, 40 μg/ml inverted bacterial membrane vesicles, 2 mM (saturating) DTT and variable concentrations of ATPαS, ADP (typically 50–100 μM) and total phosphate (typically 10 mM). The luminescence was monitored with a Promega GloMax luminometer in steps of 30 s, allowing additions of relevant reagents to the tube when required.

The baseline was first measured (30 s), before 250 pmol ATP were added as a calibration standard (30 s). Ubiquinone Q_1_, an ethanol soluble analogue of ubiquinone that can be reduced by DTT, was used to energize the membrane by *bo3* oxidase proton pumping and start the ATP synthesis (3 × 30 s). ATP synthesis could be interrupted by the addition of 400 nM potassium cyanide or 10 μM CCCP (2 × 30 s).

## Supplementary information

Supplementary Information
