# Supplementary material for: ATP synthesis at physiological nucleotide concentrations
Source: Sci Rep. 2019 Feb 28;9:3070. doi: 10.1038/s41598-019-38564-0 (PMC6395684; doi:10.1038/s41598-019-38564-0)
Supplement: Supplementary file 1 — Supplementary Information [file 41598_2019_38564_MOESM1_ESM.pdf]

Supplementary information

ATP synthesis at physiological nucleotide concentrations

Axel Meyrat and Christoph von Ballmoos

Department of Chemistry and Biochemistry, University of Bern, Freiestrasse 3, 3012 Bern, Switzerland

Correspondence: christoph.vonballmoos@dcb.unibe.ch

Supplementary Table 1:

	ATP (nmol mg^{-1} min^{-1})		
	80 µM UQ_1		500 µM NADH
	- CCCP	+ CCCP	- CCCP
BL21	25	3	15
DK8:pBWU13	44	8	22

Luciferin/luciferase ATP synthesis assay comparing the IMVs from the strains BL21 and DK8:pBWU13, and the electron donors DTT/Q_1 (80 µM UQ_1) and NADH (500 µM). The inhibitory effect of 10 µM CCCP was also tested.

Supplementary Figure S1:

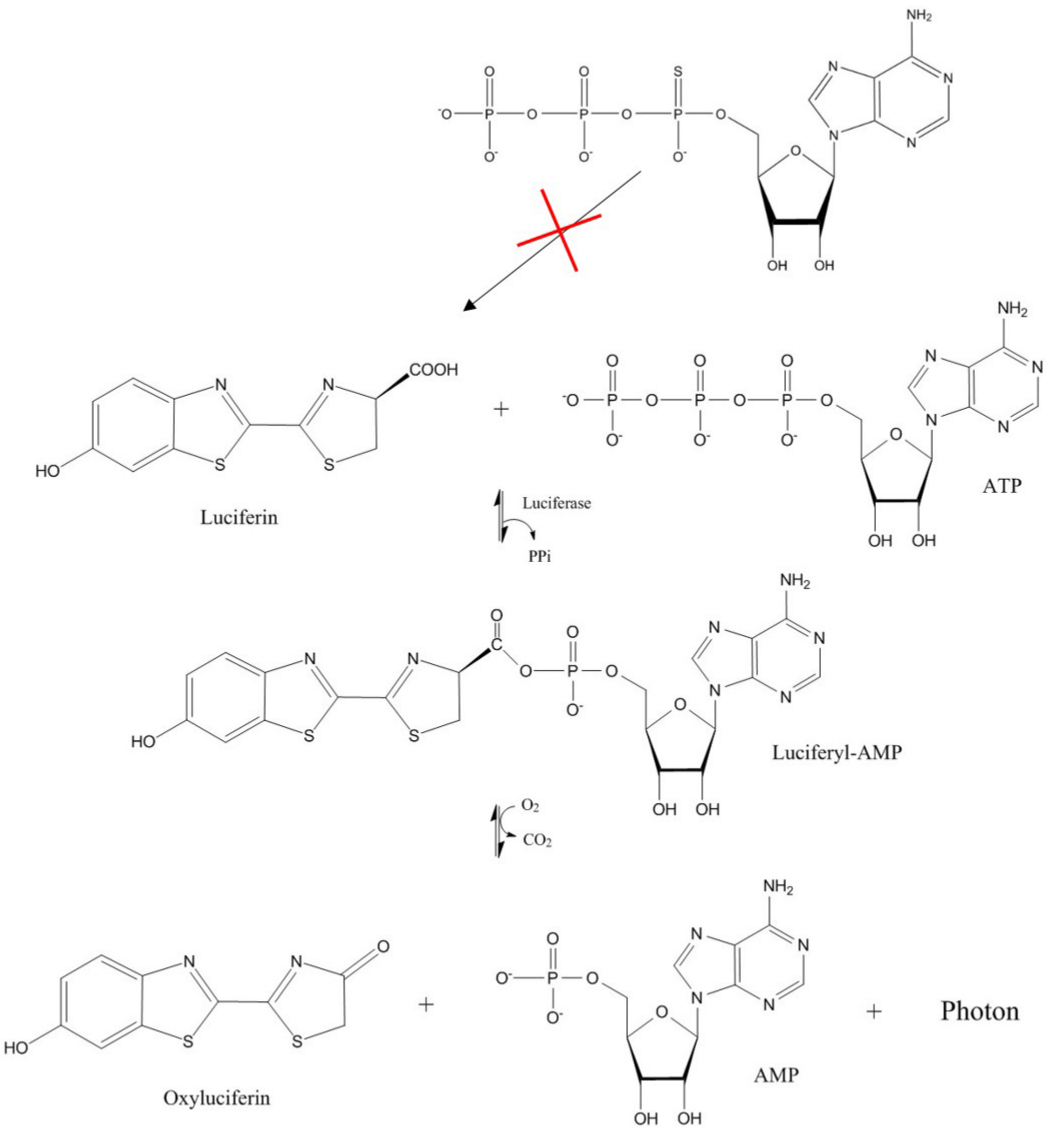

Overview of the chemical reaction of ATP with luciferin/luciferase (adapted from [25]). Mg^{2+}, an essential cofactor, is not shown. The carboxyl group of luciferin binds to the α-phosphate of ATP and the resulting compound, luciferyl-AMP, is able to oxidize dioxygen to form oxyluciferin in an excited state. AMP is formed in the process, and oxyluciferin releases a photon. ATPαS, containing a sulfur on the α-phosphate that prevents binding of luciferin, is shown on top.

Supplementary Figure S2:

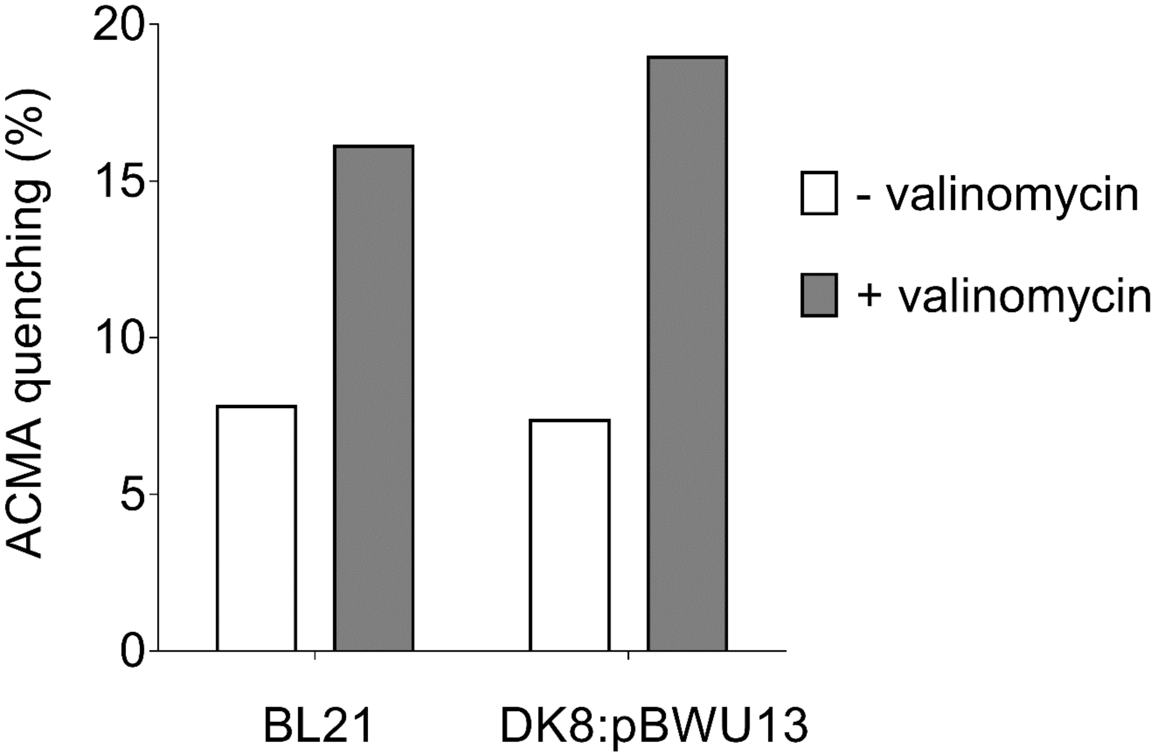

Initial ACMA quenching (within 3 sec of ATP addition) of BL21 and DK8:pBWU13 150 µg/ml IMVs in the absence (open bars) or presence (black bars) of 1 µM valinomycin, upon addition of 500 µM ATP. Shown are the average values from two independent measurements.

Supplementary Figure S3:

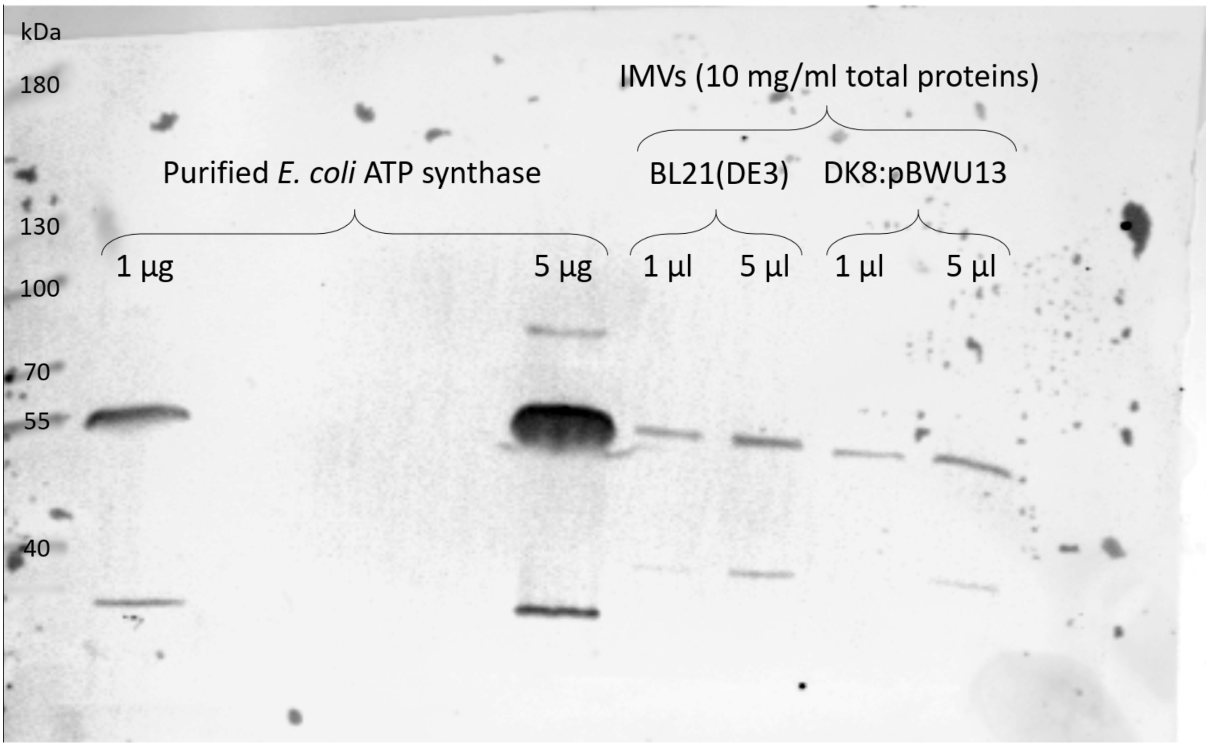

Western blot of BL21(DE3)pLysS vs. DK8:pBWU13 inverted membrane vesicles, using anti-β ATPase subunit rabbit antibodies (Agrisera) and anti-rabbit DyLight 800 (Rockland) secondary antibodies. Purified ATP synthase (ATPS) was loaded for comparison.

Supplementary Figure S4:

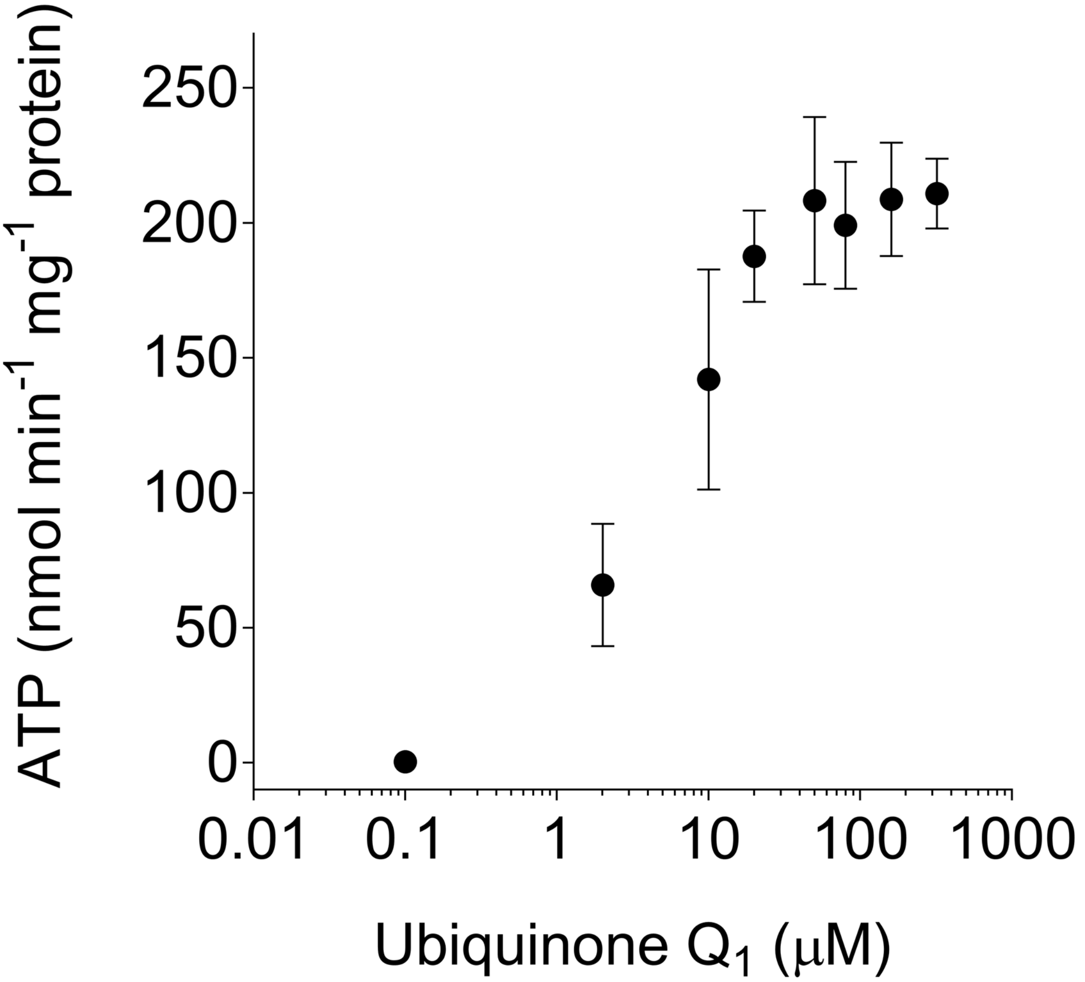

Titration of ubiquinone Q_1 for ATP synthesis. Ubiquinone Q_1 is an analog of ubiquinone which reacts with the terminal respiratory enzyme cytochrome *bo*$_3$ oxidase. The ATP synthesis rate reached a maximum of 208 ± 31 nmol ATP min^{-1} mg^{-1} total membrane proteins at 50 µM Q_1 (apparent K_m = 4.5 µM). Shown are the average and the standard deviation from three measurements.

Supplementary Figure S5:

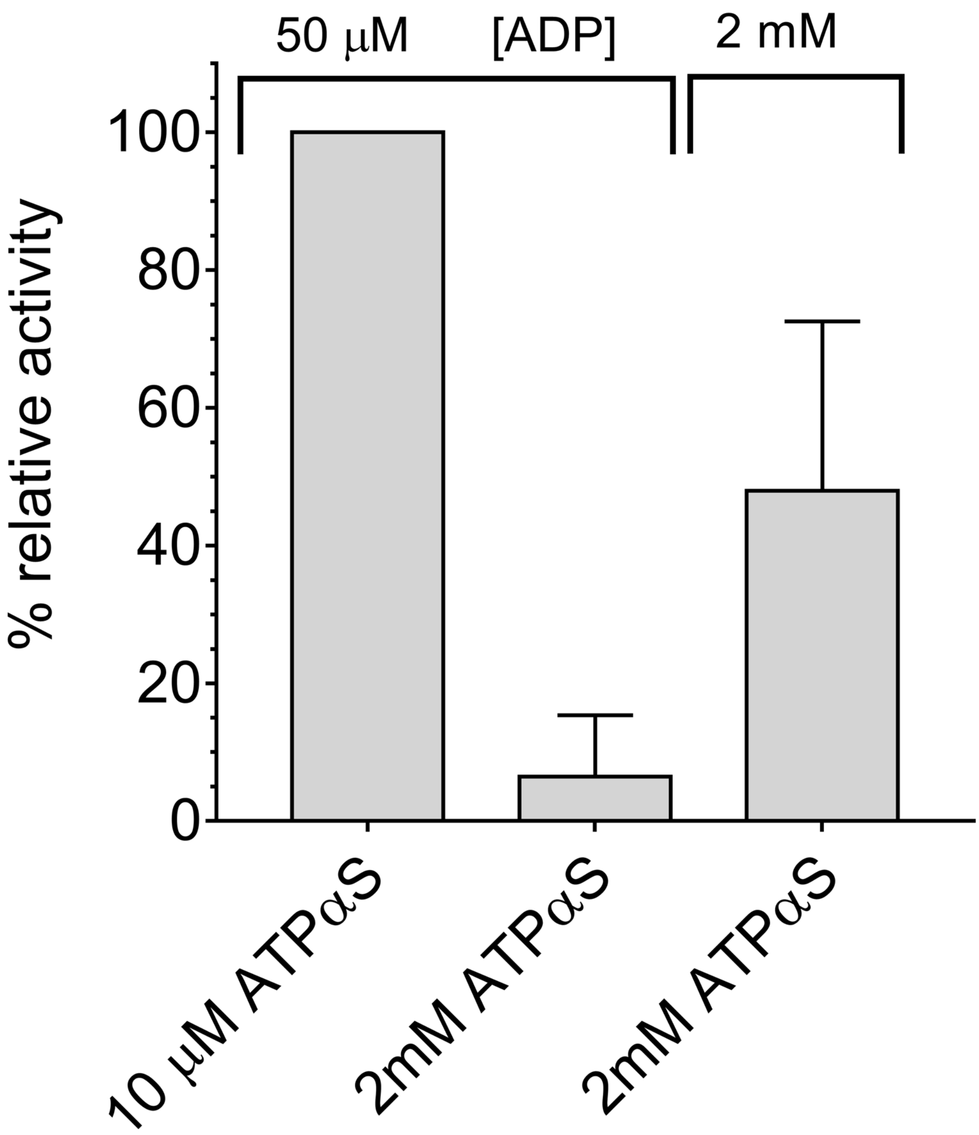

Effect of different ATPαS/ADP ratios on the ATP synthesis rate of proteoliposomes containing purified *E. coli* F_0F_1 ATP synthase and *bo₃*quinol oxidase. The activity was measured with the luciferin/luciferase ATP synthesis assay as described in material and methods, except that 1mg/ml luciferase reagent was used. Shown are the average values of two different co-reconstitution experiments at the indicated nucleotide concentrations (total *n*=5).
